# Does any fish scale of a fish have the same number of marks? A case study for two Mugilidae species

**DOI:** 10.1111/jfb.70308

**Published:** 2025-12-22

**Authors:** Ebenecer Guerra, Arturo Bell Enríquez‐García, Víctor H. Cruz‐Escalona, Eloísa Pacheco‐Almanzar, Sergio Álvarez‐Hernández, Emigdio Marín‐Enríquez, Ana Laura Ibáñez

**Affiliations:** ^1^ Universidad Autónoma Metropolitana‐Iztapalapa Departamento de Hidrobiología Alcaldía Iztapalapa Mexico; ^2^ Centro Interdisciplinario de Ciencias Marinas del Instituto Politécnico Nacional La Paz Mexico; ^3^ SECIHTI – Facultad de Ciencias del Mar, Universidad Autónoma de Sinaloa Mazatlán Mexico

**Keywords:** age determination, growth rings, Gulf of Mexico, lepidology, *Mugil cephalus*, *Mugil curema*, mullet

## Abstract

This study evaluates the difference in growth marks in scales from nine body areas of two Mugilidae species from the Gulf of Mexico: *Mugil curema* and *Mugil cephalus*. It addresses whether the different body areas show more (or fewer) marks, and which area(s) would be more useful in fish biology studies relying on mark analysis. We hypothesized no differences in the number of marks, neither across body areas nor between sexes. We counted the marks in 3704 and 6374 scales from 117 and 200 adult specimens of *M. cephalus* and *M. curema*, respectively. Although we did not count the marks on damaged or regenerated scales, we obtained their percentage of occurrence. We decided to discard the effect of the sampling location on the number of marks using a Kruskal–Wallis test (*p* = 0.073). The number of scale marks in the different body areas was modelled as a function of sex and body area using hierarchical Bayesian zero‐inflated Poisson models. The highest percentages of regenerated or damaged scales were found in the anterior areas of *M. curema* and the ventral‐anterior area of *M. cephalus*. We found a differential effect of both sex and body area on the number of marks. For *M. cephalus*, only the sex did not have a probable effect. The number of marks decreased from the anterior‐to‐posterior areas along the middle area, whereas the ventral and dorsal areas showed the opposite trend. Therefore, the central area was the least variable. For *M. curema*, males had more marks than females, the ventral area exhibited more marks than the others, whereas the middle area was less variable. Based on these results, we suggest that scales from the middle‐central area are more suitable for age studies for both species.

## 1 INTRODUCTION

Fish scales are calcified structures widely used in fish age determination and growth estimation. Compared to other structures, such as otoliths, they have the advantages of low costs, ease of large sample examination, non‐invasiveness and avoiding sacrificing the animal (Bagenal, 1974).

The scale is formed from a central zone called the focus, around which concentric layers of sclerites deposit, forming concentric striations called *circuli*. The formation of annual marks (*annuli*) on the scales results from physiological and metabolic changes caused by cyclic events or seasonal environmental changes during an annual period (Everhart & Youngs, 1981; Miranda & Escala, 2000; Panfili et al., 2002). For this reason, growth discontinuities in the scales record the organism's growth throughout its different life phases, possibly reflecting environmental conditions, feeding periods or migrations, thus allowing the extraction of specific chronological information in the fish's life, creating methodological options for stock identification and estimates of organism's growth and age (Brophy, 2014; Panfili et al., 2002).

In most fishes, the first scale appears on the caudal area's midline followed by a rapid extension to the row's front and back, whereas new rows appear dorsally and ventrally (Sire & Akimenko, 2004). The developing scales are juxtaposed at the beginning, becoming increasingly captive and covered by each other as they increase in diameter, creating a checkerboard‐like growth pattern with a pre‐established scaling pattern. The first scales in *Mugil cephalus* appear in larvae of ~6.4 mm standard length (SL), and the development of squamation begins between the second dorsal fin and the anal fin at the height of the horizontal septum. The horizontal scale rows are developed in anterior and posterior directions; more scales are added dorsally and ventrally shortly after (Thieme et al., 2021). The squamation of *Mugil saliens* follows a similar pattern (Burdak, 1969).

Morphological data suggest that there is a predetermined scaling pattern prior to scale morphogenesis, possibly determined by the sonic hedgehog protein (SHH), which plays an essential role in the regulation of vertebrate organogenesis; however, to date, there is no explanation of the factors that determine the formation and development of scales in that order (Levin, 2011; Sire & Akimenko, 2004).

Regardless of the widespread use of scales in fisheries biology, to our knowledge, there are no studies or research that address whether there are differences in the number of growth marks relative to the body area. Therefore, this study evaluates the differences in the number of growth marks in scales across nine body areas of two Mugilidae species from the Gulf of Mexico: *Mugil curema* and *M. cephalus*. It addresses whether the different body areas show more (or fewer) marks, whether there are differences between sexes, controlling for scale length, and, therefore, which area(s) would be more useful in fish biology studies relying on scale mark analysis.

## 2 MATERIALS AND METHODS

### 2.1 Sample collection

**FIGURE 1:**
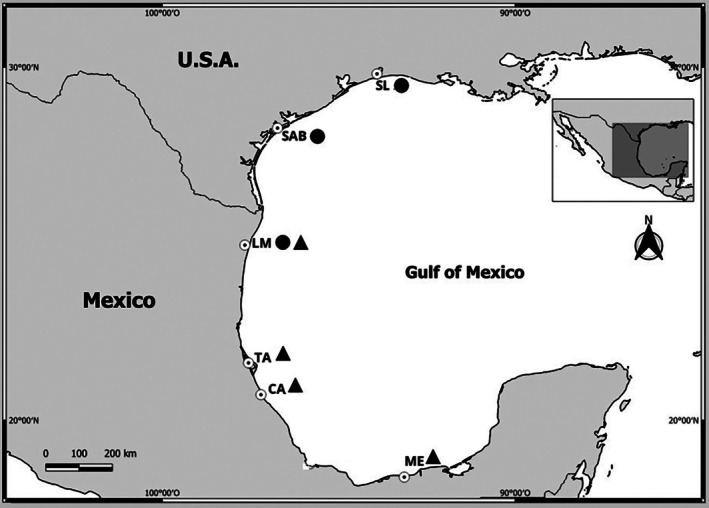
Sampling locations by species; the circles and triangles correspond to the presence of *Mugil cephalus* and *Mugil curema*, respectively. SL, Sabine Lake Texas, USA; SAB, San Antonio Bay, Texas, USA; LM, Madre Lagoon, Tamaulipas; TA, Tamiahua Lagoon, Veracruz; CA, Cazones River, Veracruz; ME, Mecoacán Lagoon, Tabasco.

Specimens of *M. cephalus* (*n* = 117) and *M. curema* (*n* = 200) were collected by commercial fishers from 2008 to 2009 at different locations along the Gulf of Mexico (Figure 1). The localities for *M. cephalus* were Sabine Lake and San Antonio Bay, Texas, Madre Lagoon, Tamaulipas (Table 1). For *M. curema*, the localities were Madre Lagoon, Tamaulipas, Tamiahua Lagoon and Cazones River, Veracruz, and Mecoacan Lagoon, Tabasco (Table 1).

**TABLE 1:** Characteristics of *Mugil cephalus* and *Mugil curema* specimens: locations, group code, number of specimens (*N*), number of scales (*n*), sex, collection date and standard length (cm).

Locations	Group code	*N*	*n*	Sex		Collection date	Standard length	
				Female	Male		Range	Mean ± SD
*Mugil cephalus*								
Sabine Lake, Texas, USA	SL	50	1416	37	13	Sep 2009	24.0–41.0	27.0 ± 3.5
San Antonio Bay, Texas, USA	SAB	22	661	13	9	Sep 2009	24.0–33.5	27.4 ± 2.9
Madre Lagoon, Tamaulipas, Mexico	LM	45	1627	14	31	Sep 2009	21.5–28.3	24.3 ± 1.7
*Mugil curema*								
Madre Lagoon, Tamaulipas, Mexico	LM	51	1593	43	8	Jan 2009	19.5–25.0	22.4 ± 1.0
Tamiahua Lagoon, Veracruz, Mexico	TA	46	1494	42	4	Jan 2009	21.2–27.7	23.8 ± 1.5
Cazones River, Veracruz, Mexico	CA	50	1599	42	8	Feb 2009	21.8–31.0	24.3 ± 1.6
Mecoacán Lagoon, Tabasco, Mexico	ME	53	1688	26	27	Nov 2008	17.4–32.2	22.5 ± 3.4

**FIGURE 2:**
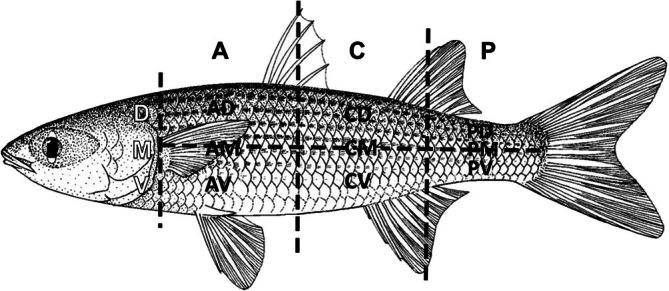
Areas where scales were sampled. A, anterior; C, central; P, posterior; D, dorsal; M, middle; V, ventral. Figure of the fish is from Harrison (2002).

Taxonomic species identification followed the FAO key by Harrison (2002). Individuals were measured for total length (TL) and standard length (SL)] and were sexed visually. The body's lateral area was divided into sections following Ibáñez et al. (2009). Longitudinally, the areas were anterior (A), from the end of the operculum to the middle of the dorsal fin; central (C), from the middle of the first dorsal fin to the middle of the second dorsal fin; posterior (P), between the middle of the second dorsal fin at the beginning of the caudal fin. Transversely, the fish's body was divided into three areas: middle (M), corresponding to the level of the fish's lateral line, and dorsal (D) and ventral (V), corresponding to the upper and lower areas of the lateral line, respectively. Thus, the fish's left side was divided into nine areas: AD, AM, AV, CD, CM, CV, PD, PM and PV (Figure 2).

**FIGURE 3:**
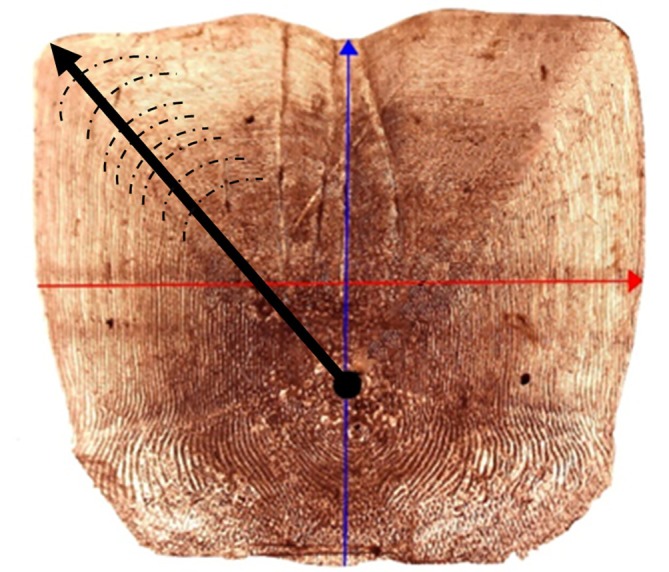
Fish scale from *Mugil cephalus;* the blue line corresponds to the length of the scale (Lsc); the red line corresponds to the width of the scale (Wsc); the black dot shows the focus; the solid black line indicates the axis of the radius (R) from the focus to the posterior margin of the scale, whereas the black dotted lines correspond to examples of growth rings.

Three to four scales were collected from each area. Each scale was cleaned with soap and water and stored dried. The scales were mounted between two microscope slides and photographed from a zenithal plane using a digital microscope (CELESTRON Microcapture Pro). Each scale's length (Lsc), width (Wsc) and radius (R) (Figure 3) were measured using the free image processor ImageJ‐FIJI (Schindelin et al., 2012).

The number of growth marks in ctenoid scales was determined following Joseph (1962). This process was performed on the scales in each specimen's nine body areas.

Scales were read by a single reader with intensive training beforehand advised by one of the authors who has extensive experience in reading scales. During the training, the first author read the scales over 3 months of training and, later, during the pandemic, did all the readings that were done only once due to pandemic closure. A dataset was created with the number of marks in the scales of each area and specimen. Damaged and regenerated scales were not analysed because abnormal scale development may alter the number of marks. However, the ratio of scales in good status to damaged and regenerated scales was obtained by area and locality for each species.

### 2.2 Data analysis

The final dataset consisted of scale mark counts obtained from the nine body sections of *M. cephalus* and *M. curema*, the scale length and status (good, damaged or recovered), the individual's sex and the sampling locality. We tested for differences in scale marks by localities using a non‐parametric Kruskal–Wallis test, for which we did not reject the null hypothesis (*p* = 0.073); that is, there were no significant differences between localities, and thus, the factor was not considered in subsequent analyses.

Given that the replicates are at an individual level, and the different body sections reflect repeated measures, we analysed the effects of the different factors using a Bayesian non‐centred hierarchical Poisson model per species. Bayesian inference updates the credibility of model parameters based on prior knowledge about them and the supplied data (van de Schoot et al., 2021). Bayesian hierarchical models, on the contrary, are random effects models that incorporate the uncertainty surrounding the parameters at the lower levels of the hierarchy and sequentially transfer it to the higher (shared) levels; therefore, they effectively allow the investigation of cross‐level hypotheses and minimize the effect of data imbalances (Gelman et al., 2014). In our case, the individuals form the lower level of the hierarchy, accounting for the repeated‐measures design, and the species form the higher level. We chose a non‐centred approach due to the many groups (individuals) in the lower‐level hierarchy. Efficiently sampling posterior distributions in Bayesian models (and thus unbiased estimates) depends on the geometric ergodicity between a Markov transition and the target distribution; however, the hierarchies give place to a significant curvature, otherwise known as the hierarchical funnel (Betancourt, 2017): the group‐level variances are restricted within the shared variances. In a traditional hierarchical model, the group‐level parameters are centred around the shared mean with shared variance, which controls the amount of shrinkage. In a non‐centred approach, the group‐level parameters are estimated indirectly using an offset (the difference between each group's mean and the shared mean) and scaled back using the shared variance. Thus, the model was specified as follows, where i represents the individual and f the factors:

- Shared priors:
  - $\alpha ∼\text{Normal}\left(\mu =0,\sigma =10\right)$: species‐level intercept, representing the mean number of marks without considering other factors.
  - $\alpha_{\sigma }∼\text{HalfCauchy}\left(\beta =5\right)$: shrinkage factor for the individual‐level intercepts.
  - $\beta_{f}∼\text{Normal}\left(\mu =0,\sigma =10\right)$: species‐level effect of each factor.
- Individual‐level priors:
  - $\Delta \alpha_{i}∼\text{Normal}\left(\mu =0,\sigma =1\right)$: the offset between each individual's and the species‐level intercepts.
  - $\alpha_{i}=\alpha +\Delta \alpha_{i}*\alpha_{\sigma }$: individual‐level intercepts, recovered from adding the species‐level intercept and the individual's offset scaled by the shrinkage factor.
  - $\Delta \beta_{i,f}∼\text{Normal}\left(\mu =0,\sigma =1\right)$: the offset between each individual's and the species‐level slopes.
  - $\beta_{i,f}=\beta_{f}+\Delta \beta_{i,f}*\beta \sigma_{f}$: effect of the factors at the individual level, recovered in the same way as the intercepts.
- Linear predictor and exponential inverse link function:
  - $\lambda =e^{\alpha_{i}+X\cdot \beta_{i,f}}$
- Likelihood: a Poisson distribution to model the average number of marks in a scale (λ):
  - $\hat{N}∼\text{Poisson}\left(\lambda =\lambda \right)$

Because growth marks and individual size are correlated, the scale length was included in the model as a control variable. We chose the scale length over the standard length because there is a high correlation between both measurements (Pearson's *r* = 0.83 and 0.6 for *M. cephalus* and *M. curema*, respectively; Figure S1) and, more importantly, because it acts as a scale‐level control variable, rather than the individual‐level standard length, better reflecting our dataset.

We implemented one model per species using the PyMC module (version 5.8.2, Abril‐Pla et al., 2023) in Python (version 3.11.5, van Rossum & Drake, 2009). The posterior distributions were sampled with four Markov chain Monte‐Carlo algorithms using a Hamiltonian sampler [No‐U‐Turn Sampler (NUTS)]. Chains were run until convergence, that is, zero divergences during the posterior sampling (Betancourt, 2017), potential scale reduction factors (Gelman‐Rubin statistics) below 1.01 and effective sample sizes greater than 2000 for all parameters. The models' goodness‐of‐fit was also verified using other graphical diagnostics such as posterior predictive checks, energy plots (Gabry et al., 2017) and Bayesian ‘*p*‐values’ close to 0.5 (Gelman et al., 2014). The posterior distributions are summarized in terms of their means and 95% highest density intervals ($\mathrm{HD}I_{95\%}$), which represent the areas of highest probability for the true value of a parameter, given the data and the model (Bolstad, 2004; Kruschke, 2015).

The effects were evaluated in terms of their size (after removing the inverse exponential link) and their probability of being greater or smaller than 1. Lastly, the posterior samples were imported into R (version 4.3.2, R Core Team, 2023) and plotted using the ggplot2 (version 3.4.4, Wickham, 2016) and ggridges (version 0.5.6, Wilke, 2024) packages.

This research did not require ethical statement approval because both species are exploited commercially along both coasts of Mexico.

## 3 RESULTS

The highest percentages of regenerated or damaged scales for *M. curema* were found in the anterior areas across all localities (i.e., Madre, Tamiahua and Mecoacán Lagoons, Cazones River Sabine Lake and San Antonio Bay); for *M. cephalus*, the highest percentages were found in the AV and CV areas (Table 2).

**TABLE 2:** Percentage of regenerated or damaged scales by area of the body and by localities for *Mugil curema* and *Mugil cephalus*.

	*Mugil curema*				*Mugil cephalus*		
Area	Madre Lagoon	Tamiahua Lagoon	Cazones River	Mecoacan Lagoon	Sabine Lake	San Antonio Bay	Madre Lagoon
AD	21.7	9.4	23.1	**21.7**	14.9	23.6	23.6
AM	27.2	**15.6**	27.4	20.4	*9.7*	7.8	16.0
AV	**30.7**	8.7	**28.0**	*14.1*	23.2	34.7	**37.3**
CD	20.5	9.7	21.1	14.6	10.2	21.6	23.8
CM	22.3	15.5	22.3	20.9	16.6	14.1	17.3
CV	25.6	11.7	20.6	17.8	**28.8**	**42.9**	31.9
PD	20.1	*6.4*	*16.6*	18.5	16.7	14.3	16.9
PM	21.2	8.3	27.3	20.1	14.7	*6.0*	*10.2*
PV	*17.3*	11.4	20.0	15.9	19.9	9.6	14.5
Mean ± SD	23.0 ± 4.1	10.7 ± 3.1	22.9 ± 3.9	18.2 ± 2.8	17.2 ± 6.1	19.4 ± 12.6	21.3 ± 8.8

*Note*: The highest values are presented in bold, and the lowest ones are presented in italics. Areas are as shown in Figure 2.

**FIGURE 4:**
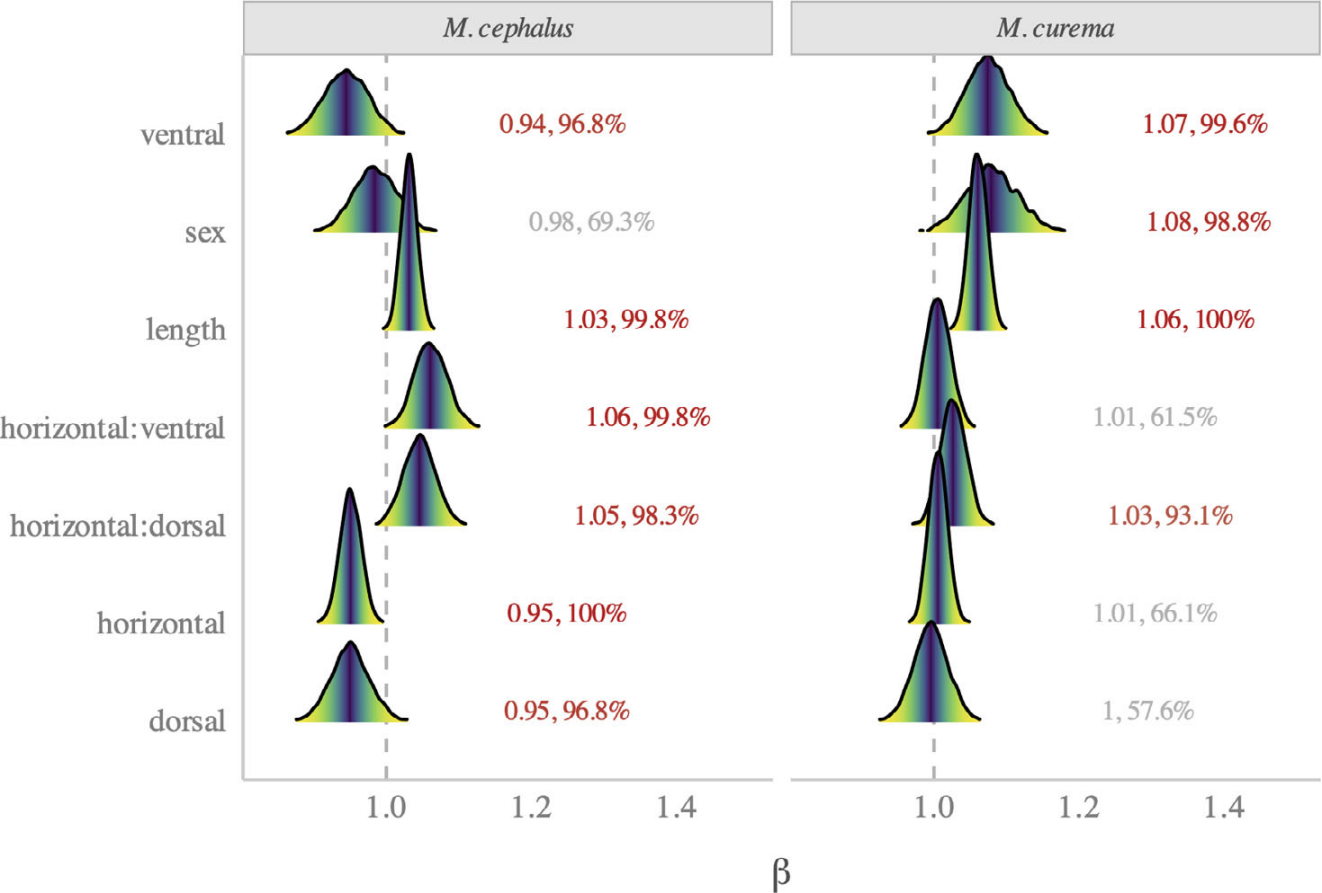
Ridgeline plots with the posterior distributions of the effects of the different factors. The vertical line at 1 indicates a null effect, whereas the text labels show the mean, $P\left(\beta \right)\neq 1$.

Individuals from *M. cephalus* had a higher number of scale marks (median = 4, SD = 1.13) than those from *M. curema* (median = 2, SD = 0.8). As for the effects of the remaining explanatory factors on the number of marks (sex, scale length and body sections), the species‐level coefficients are shown without the exponential inverse link to allow for a direct interpretation and are presented in Figure 4. A table with full numerical summaries is available in the supplemental information SI.

Without considering any explanatory factors, scales from *M. cephalus* had a higher mean number of marks [α=5.75±0.59, (4.62, 6.87)] than *M. curema* [α=3.37±0.35, (2.72, 4.05)]. The effects of the factors varied between species.

**FIGURE 5:**
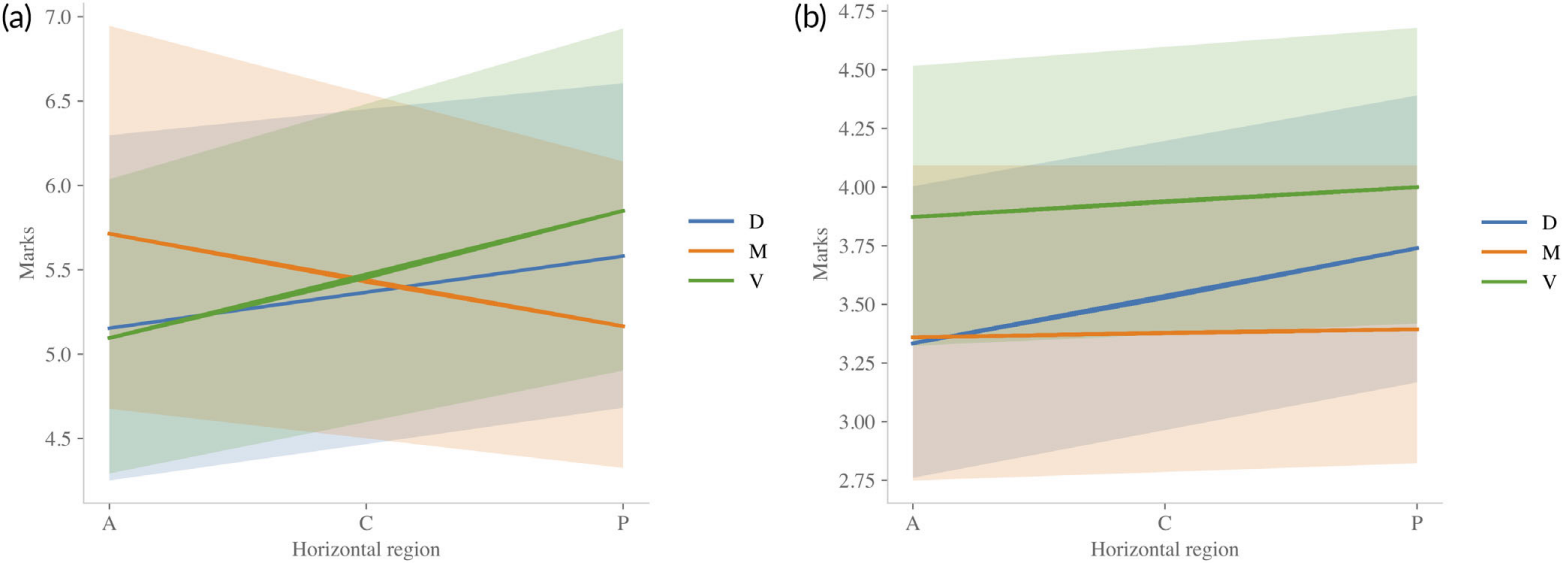
Interaction between the central tendency for horizontal and vertical body sections. Dorsal = blue; medium = orange; ventral = green. Ribbons: 95% highest‐density intervals. (a) *Mugil cephalus*; (b) *Mugil curema*.

First, for *M. cephalus*, most factors had relatively small (within 5%–10%), albeit highly probable, effects $[P\left(\beta \neq 1\right)>96\%$], except for sex ($\beta_{\mathrm{sex}}\approx 0.98;P\approx 69\%$). As for the body areas, although the main effects were highly probable, the interaction effects show that the number of marks along the horizontal areas depends on the vertical section: along the M area, they decrease A–P, whereas both the ventral and dorsal areas show the opposite trend (Figure 5a). Thus, the central area shows the smallest difference in the number of scale marks.

For *M. curema*, in contrast, males had 7% more marks than females. Regarding the other factors, only scale length, scales from the ventral areas and the combination of horizontal and dorsal areas showed highly exploratory effects. The latter, however, had a relatively negligible effect (3%) on the number of scale marks. The number of marks increased by 6% per unit increase in scale length [$P\left(\beta >1\right)=100$]. Finally, a scale from the ventral areas had, on average, 7% more marks than the middle area.

In summary, we found that the number of marks differed based on sex only in *M. curema*, whereas, as expected, the scale length positively influenced the number of marks for both species. Regarding the body areas, in *M. cephalus*, the number of marks among the vertical areas was similar along the C area and thus should be preferred in future studies. For *M. curema*, on the contrary, the V area had more marks than the others, whereas the M area showed virtually no differences among horizontal body sections (Figure 5b); therefore, the latter should be used for analysis.

## 4 DISCUSSION

### 4.1 Differences in marks in scales extracted from different body areas

The frequency of scale *circuli* formation and marks varies between species and with growth rate (Fisher & Pearcy, 1990; Friedland et al., 1993; Ibañez et al., 2008). Therefore, it was expected to find differences in the number of marks between Mugilidae species. On the contrary, we found similar ‘marking patterns’ between them.

*M. cephalus* had more marks than *M. curema*, which could be explained by the larger absolute sizes of the specimens from *M. cephalus* (standard length = 26.8 ± 3.8 cm, mean ± SD) than those from *M. curema* (22.9 ± 2.3 cm). Additionally, the growth rate is lower in *M. cephalus* (k = 0.009) than in *M. curema* (k = 0.14) (Ibáñez et al., 1999).

The P area had more marks in both species, coinciding with the previously described scaling form (Burdak, 1969; Sire & Akimenko, 2004; Thieme et al., 2021). The first scales appear in the middle caudal‐fin area, followed by a rapid extension to the front and back of this row and a simultaneous appearance of rows in the D and V areas, which could explain why those areas showed the highest number of marks as they appear first.

The central area showed the smallest difference in number of marks across vertical areas for *M. cephalus* and *M. curema*, particularly in the middle.

Fish scales form at different times depending on the body area, whereas those from older animals may thicken, become resorbed and cease or become irregular in growth (Casselman, 1990); however, the individuals we analysed are well below the reported longevity (18.7 and 28.3 years) and total length (64.1 and 46.1 cm) for *M. curema* and *M. cephalus*, respectively (Ibáñez‐Aguirre & Gallardo Cabello, 1996).

The most appropriate sites for scale removal are usually assumed to be in the latero‐dorsal area of the body, in an area where scales are protected from damage and are less likely to be affected by regeneration (e.g., under the pectoral fin; Panfili et al., 2002). However, it is also suggested that initial investigations of scale growth marks for a particular species should include a comparative analysis of scales from different body locations (Panfili et al., 2002).

We found that the best area to extract scales for either species is the central‐middle area, due to the small differences in the number of marks compared to the other body areas and the lower abundance of damaged or regenerated scales relative to the anterior areas.

### 4.2 Using scales versus other hard structures on age and growth studies

For fish of the Mugilidae family, ageing studies that compared scales versus otoliths have yielded very similar results, because a single annulus, which is deposited once a year, has been observed in both structures (Hsu & Tzeng, 2009). However, for other fish species, using scales to determine a fish age might not be the best method, although using scales provides some advantages compared to studies using other bony structures, such as otoliths and spines. The most straightforward advantage is the ability to perform ageing studies using live individuals, because the extraction of scales does not require killing of the animals. Using scales to determine age has been successfully used in aquaculture experiments for the carp *Labeo rohita* (Eknath & Doyle, 1985) and for wild young largemouth bass *Micropterus salmoides* (Morehouse et al., 2013). Moreover, Cheung et al. (2007) used scales to assess growth impairment of three fish species under different culture conditions.

Using scales is also more cost‐effective, because there is no special treatment prior to reading (Hsu & Tzeng, 2009). Using otoliths, on the contrary, is costlier and requires more time, because otoliths need to be prepared (e.g., sectioned, dyed, etc.) before reading, and the different techniques used for preparation can influence the otolith's optical properties, and thus have an effect in the estimation of age (Winkler et al., 2019). Nonetheless, we found that the difference in the number of scale marks among body areas is larger in *M. cephalus* than *M. curema*; therefore, species‐specific studies comparing age estimates from different body areas to those from otoliths are needed, as well as research evaluating the potential implications for fisheries management.

We recommend extracting scales from the central‐middle area in both *M. cephalus* and *M. curema*. Moreover, using scales to determine fish age is a simpler and more cost‐effective method that can be used in live individuals, albeit some loss of accuracy should be considered compared to other bony structures.

## AUTHOR CONTRIBUTIONS

**Ebenecer Guerra:** data curation, formal analysis, investigation, methodology, visualization, writing – original draft, writing – review and editing. **Arturo Bell Enríquez‐García, Emigdio Marín‐Enriquez and Sergio Álvarez‐Hernández:** formal analysis, investigation, methodology, visualization – review and editing. **Víctor H. Cruz‐Escalona and Eloísa Pacheco‐Almanzar:** writing – review, editing and members of the study committee of the master's degree in biology. **Ana L. Ibáñez:** conceptualization, project administration, resources, supervision, writing – review and editing. All authors read and approved the final manuscript.

## FUNDING INFORMATION

The study was funded by grants from the Universidad Autónoma Metropolitana‐Iztapalapa and Secretaría de Educación Pública‐Consejo Nacional de Ciencia y Tecnología (Ciencia Básica: 2011‐01‐165569).

Ebenecer Guerra made the data curation, formal analysis, investigation and writing of the original draft. Arturo Bell Enríquez‐García, Emigdio Marín‐Enriquez and Sergio Álvarez‐Hernández made the formal analysis, review and editing. Víctor H. Cruz‐Escalona and Eloísa Pacheco‐Almanzar did review and editing. Ana L. Ibáñez made the conceptualization, project administration, writing and review. All authors read and approved the final manuscript.

## CONFLICT OF INTEREST STATEMENT

All authors certify that they have no affiliations with, or involvement in, any organization or entity with any financial or non‐financial interest in the subject matter or materials discussed in this manuscript.

## Supporting information


**Table S1.** Numerical summaries of the posterior distributions of the hierarchical Bayesian models for the number of marks on scales from different body sections of *Mugil cephalus* and *Mugil curema*. ESS, effective sample size; HDI, highest‐density interval; Rhat, potential scale‐reduction factor.

## Data Availability

The datasets generated and/or analysed during the current study are available from the corresponding author on reasonable request.
