# Supplementary material for: Does any fish scale of a fish have the same number of marks? A case study for two Mugilidae species
Source: J Fish Biol. 2025 Dec 22;108(4):1276–83. doi: 10.1111/jfb.70308 (PMC13193495; doi:10.1111/jfb.70308)
Supplement: Supplementary file 1 — Table S1. Numerical summaries of the posterior distributions of the hierarchical Bayesian models for the number of marks on scales from different body sections of Mugil cephalus and Mugil curema. ESS, effective sample size; HDI, highest‐density interval; Rhat, potential scale‐reduction factor. [file JFB-108-1276-s001.docx]

**Supplemental Information**

**Table S1** Numerical summaries of the posterior distributions of the hierarchical Bayesian models for the number of marks on scales from different body sections of *Mugil cephalus* and *Mugil curema*. ESS: effective sample size; Rhat: potential scale-reduction factor, HDI: Highest Density Interval.

|  | **Mean** | **SD** | **95% HDI** | **ESS** | **Rhat** |
| --- | --- | --- | --- | --- | --- |
| *Mugil cephalus* |  |  |  |  |  |
| Α | 5.75 | 0.59 | [4.62, 6.87] | 2440 | 1 |
| β[length] | 1.03 | 0.01 | [1.01, 1.05] | 2684 | 1 |
| β[sex] | 0.98 | 0.03 | [0.93, 1.04] | 3513 | 1 |
| β[dorsal] | 0.95 | 0.03 | [0.90, 1.00] | 2667 | 1 |
| β[ventral] | 0.94 | 0.03 | [0.89, 1.01] | 2730 | 1 |
| β[horizontal] | 0.95 | 0.01 | [0.92, 0.98] | 2376 | 1 |
| β[horizontal:ventral] | 1.06 | 0.02 | [1.02, 1.11] | 2931 | 1 |
| β[horizontal:dorsal] | 1.05 | 0.02 | [1.00, 1.09] | 3072 | 1 |
| *Mugil curema* |  |  |  |  |  |
| Α | 3.37 | 0.35 | [2.72, 4.05] | 2638 | 1 |
| β[length] | 1.06 | 0.01 | [1.04, 1.08] | 2817 | 1 |
| β[sex] | 1.06 | 0.04 | [1.01, 1.15] | 3103 | 1 |
| β[dorsal] | 1.00 | 0.02 | [0.95, 1.04] | 4011 | 1 |
| β[ventral] | 1.07 | 0.03 | [1.01, 1.13] | 3022 | 1 |
| β[horizontal] | 1.01 | 0.01 | [0.98, 1.03] | 3660 | 1 |
| β[horizontal:ventral] | 1.01 | 0.02 | [0.97, 1.04] | 4472 | 1 |
| β[horizontal:dorsal] | 1.03 | 0.02 | [0.99, 1.06] | 4690 | 1 |
